# Comparison of acute treatment with delayed-onset versus rapid-acting antidepressants on effort-related choice behaviour

**DOI:** 10.1007/s00213-020-05541-9

**Published:** 2020-05-20

**Authors:** Simonas Griesius, Jack R Mellor, Emma SJ Robinson

**Affiliations:** School of Physiology, Pharmacology and Neuroscience, University of Bristol, Biomedical Sciences Building, University Walk, Bristol, BS8 1TD UK

**Keywords:** Major depressive disorder, Antidepressant, Motivation, Monoamine, Ketamine, Rat, Anhedonia, Rapid-acting antidepressant, Reward, Decision-making

## Abstract

**Rationale:**

Reward-related impairments are common in major depressive disorder (MDD) and may contribute to the loss of interest in pleasurable activities. A novel approach to studying reward-related decision-making are effort-based tasks; however, direct comparisons between delayed-onset and rapid-acting antidepressants (ADs) have not yet been carried out.

**Objectives:**

To investigate the effects of conventional delayed-onset ADs versus rapid-acting ADs, ketamine and scopolamine, on effort-related choice behaviour.

**Methods:**

Female Lister hooded rats were trained in an operant effort for reward task (EfRT) where animals choose between working for a high value-high effort reward and consuming low value-low effort chow. Using a within-subject study design, animals were then tested following acute treatment with different monoaminergic ADs, and the rapid-acting ADs ketamine or scopolamine.

**Results:**

Consistent with previous findings, we found choice behaviour was sensitive to dopaminergic manipulations. We observed that pre-feeding altered choice behaviour and that the use of high or low value reward differentially affected behaviour. Monoamine re-uptake inhibitors and rapid-acting ADs resulted in similar, general patterns of reduced motivation without any evidence for specific effects, and we did not observe any clear differences between these classes of antidepressant.

**Conclusions:**

Motivational changes induced by dopaminergic manipulations and pre-feeding differentially affect effort choice behaviour. However, both conventional delayed-onset ADs and ketamine and scopolamine appear to have detrimental effects on motivation in this task at the higher doses tested without any evidence of specificity for effort-related choice behaviour, in contrast to their specificity in tasks which look at more cognitive aspects of reward processing.

**Electronic supplementary material:**

The online version of this article (10.1007/s00213-020-05541-9) contains supplementary material, which is available to authorized users.

## Introduction

Major depressive disorder (MDD) is one of the leading causes of disability worldwide, accounting globally for 8.2% of all years lived with disability (Ferrari et al. 2013). Individuals with MDD often experience impairments in reward seeking, motivation, and effort-related decisions (Treadway and Zald 2011; Treadway et al. 2012; Pizzagalli 2014), with anhedonia listed as one of the main MDD symptoms in the Diagnostic and Statistical Manual of Mental Disorders 5 (DSM5) (American Psychiatric Association 2013). Anhedonia is a complex symptom consisting of consummatory and motivational components (Der-Avakian and Markou 2012; Slaney et al. 2018); however, changes in reward-related behaviour in depression may be more complex, and recent human and animal studies suggest that changes in more cognitive aspects of reward processing may be relevant (Thomsen 2015; Slaney et al. 2018). The DSM5 lists the symptom of ‘loss of interest in rewarding activities’ as an important contributor to MDD which may be influenced by anhedonia or by wider deficits in reward-related behaviour (American Psychiatric Association 2013). Delayed-onset antidepressant drugs (ADs), conventionally prescribed for MDD, either lack efficacy in terms of their effects on motivational impairments or worsen the said impairments (Stenman and Lilja 2013; Fava et al. 2014; Rothschild et al. 2014). Intriguingly, ketamine, a rapid-acting AD, rapidly and robustly ameliorates anhedonia in patients with treatment-resistant depression and treatment-resistant bipolar depression (Murrough et al. 2013; Lally et al. 2014).

Animal studies use tests of deficits in reward-related behaviour as a potential method to quantify depression-related behaviour (Slattery and Cryan 2017; Slaney et al. 2018). The most commonly used method, the sucrose preference test (SPT), measures sensitivity to reward and is reliably altered in rodents exposed to chronic stress, with deficits reversed with chronic but not acute ADs (Willner 2017). There are, however, inconsistencies in the SPT findings across different models of depression (Slaney et al. 2018), and depressed humans do not show deficits in the sweet taste test (Dichter et al. 2010). Another frequently used task is the progressive ratio task, which measures effortful motivation. The progressive ratio task also produces inconsistent findings across different models of depression (Slaney et al. 2018), possibly due to the higher ratio trials also requiring more time and therefore being susceptible to delay discounting. Therefore, the SPT and progressive ratio task may not be the most appropriate translational methods to study reward-related deficits in MDD, with methods looking more broadly at reward-related behaviour better capturing the impairments in goal-directed behaviour seen in MDD (Slaney et al. 2018). These include effort-related choice tasks such as the T-maze barrier choice task (Salamone et al. 1991; Yohn et al. 2015), the PROG/chow feeding task (Randall et al. 2012), and the FR5/chow feeding task (Nunes et al. 2013; Yohn et al. 2016a). In these tasks, animals choose between high effort-high value rewards versus low effort-low value rewards with the hypothesis that depression-related behaviour reduces high effort choices. These tasks are sensitive to dopaminergic manipulations, with amphetamine treatment selectively increasing high effort choices and dopamine antagonism reducing high effort choices (Salamone et al. 1991; Bardgett et al. 2009; Randall et al. 2012). Delayed-onset ADs, fluoxetine and desipramine, have not been shown to have acute beneficial effects in normal animals in the FR5/chow feeding task (Yohn et al. 2016a). The vesicular monoamine re-uptake inhibitor tetrabenazine has been reported to cause depression in an 80-week study of patients treated for Huntington’s disease-related chorea (Frank 2009). Tetrabenazine also reduces high effort choices in rats, which can be reversed acutely with bupropion but not with typical delayed-onset ADs (Nunes et al. 2013; Yohn et al. 2016a). In line with the tetrabenazine reports, in the human effort expenditure for rewards task (EEfRT), MDD patients exhibited motivational deficits, as well as a decrease in their ability to integrate information regarding reward magnitude and reward probability (Treadway et al. 2012). These studies, when taken together, suggest that motivation can be assessed using variations of the EfRT in humans and animal models, as well as suggesting that depressed patients and animal models of depression exhibit motivational deficits in these tasks, and that delayed-onset ADs generally have no effect on motivation in these tasks.

Recently, it has been hypothesised that the depression-related phenotype in patients arises as a product of dysregulation of cognitive-emotional neural networks and the emergence of negative affective bias (Harmer et al. 2009a; Disner et al. 2011; Slaney et al. 2018). Delayed-onset ADs acutely modify affective biases in healthy volunteers (Pringle et al. 2011) and in depressed patients (Harmer et al. 2009b), although subjective improvements in mood were delayed by several weeks. Delayed-onset ADs also induced positive bias acutely in the rat affective bias task (ABT) (Stuart et al. 2013; Hinchcliffe et al. 2017). Hinchcliffe et al. (2017) have also reported comparable effects of conventional delayed-onset ADs, acute restraint stress, and acute corticosterone on affective bias across the sexes. However, seeing as depression is more common in females than in males (Kuehner 2017), female rats were chosen for the present study. Although ketamine did not produce a positive bias in the ABT, ketamine but not venlafaxine, a delayed-onset AD, attenuated negative biases induced by psychosocial stress, and benzodiazepine inverse agonist, FG7142, induced negative bias (Stuart et al. 2015). In the rat judgement bias task (JBT), developed to investigate affective biases and decision-making behaviour, ketamine induced a positive bias acutely, whereas delayed-onset ADs produced no effect (Hales et al. 2017). Ketamine also blocked N-methyl-d-aspartate (NMDA) receptor–dependent bursting in the lateral habenula, a structure involved in the pathophysiology of MDD, and the said synchronous bursting activity drove depressive behaviour in rats in the forced swim test, SPT, and the real-time place aversion task (Yang et al. 2018). In the same study, fluoxetine had no effect on either the bursting activity or the behaviour (Yang et al. 2018). These findings suggest that affective biases related to reward learning, memory, and decision-making are differentially modulated by ketamine versus delayed-onset ADs. It remains to be investigated whether the differential effects of delayed-onset versus rapid-acting ADs also involve other aspects of reward processing, such as effort-related choice behaviour.

In this study, we used an operant effort for reward task (EfRT) to investigate the effects of acute treatment with conventional delayed-onset and rapid-acting ADs to compare the effects in this task with findings from affective bias tasks (Stuart et al. 2013; Stuart et al. 2015; Hales et al. 2017; Hinchcliffe et al. 2017). We tested the effects of acute treatment across a range of doses shown to induce positive biases associated with reward-related learning and memory, or decision-making in affective bias and judgement bias tasks. In addition, we tested the effects of manipulating the dopamine system using amphetamine, haloperidol, and tetrabenazine. We also investigated the effects of pre-feeding with either the high or low value reward. Once confident the task produced results comparable with those previously reported (Randall et al. 2012; Yohn et al. 2016a), experiments involving different monoamine re-uptake inhibitor ADs and two rapid-acting ADs, ketamine and scopolamine, commenced.

## Methods

### Animals

Adult female Lister hooded rats (*n* = 16) were purchased from Charles River and housed in pairs under reverse 12-h lighting cycles (lights off at 08:15 h) and humidity- and temperature-controlled conditions. As this assay was new to our group, sample size was based on our previous work using similar treatments and doses in the affective bias test (Hinchcliffe et al. 2017). Enrichment, in the form of a wooden chewing block, cardboard tube, plastic house, and climbing rope, was present in every cage throughout the study. Bedding consisted of sawdust and paper ribbon. Rats weighed 175–200 g at arrival and were food restricted to no less than 90% of their free-feeding body weight (matched to a normal growth curve) for the duration of the study. Supplemental chow (LabDiet, PMI Nutrition International) was given to maintain moderate weight gain throughout the study. Ad libitum water was available in the home cages. Two rats developed audiogenic seizures, which are known to arise in this strain, and were killed before completion of the study. All procedures were carried out under local institutional guidelines (approved by the University of Bristol Animal Welfare and Ethical Review Board) and in accordance with the UK Animals (Scientific Procedures) Act 1986.

### Effort for reward task

#### Apparatus and training procedure

**Fig. 1:**
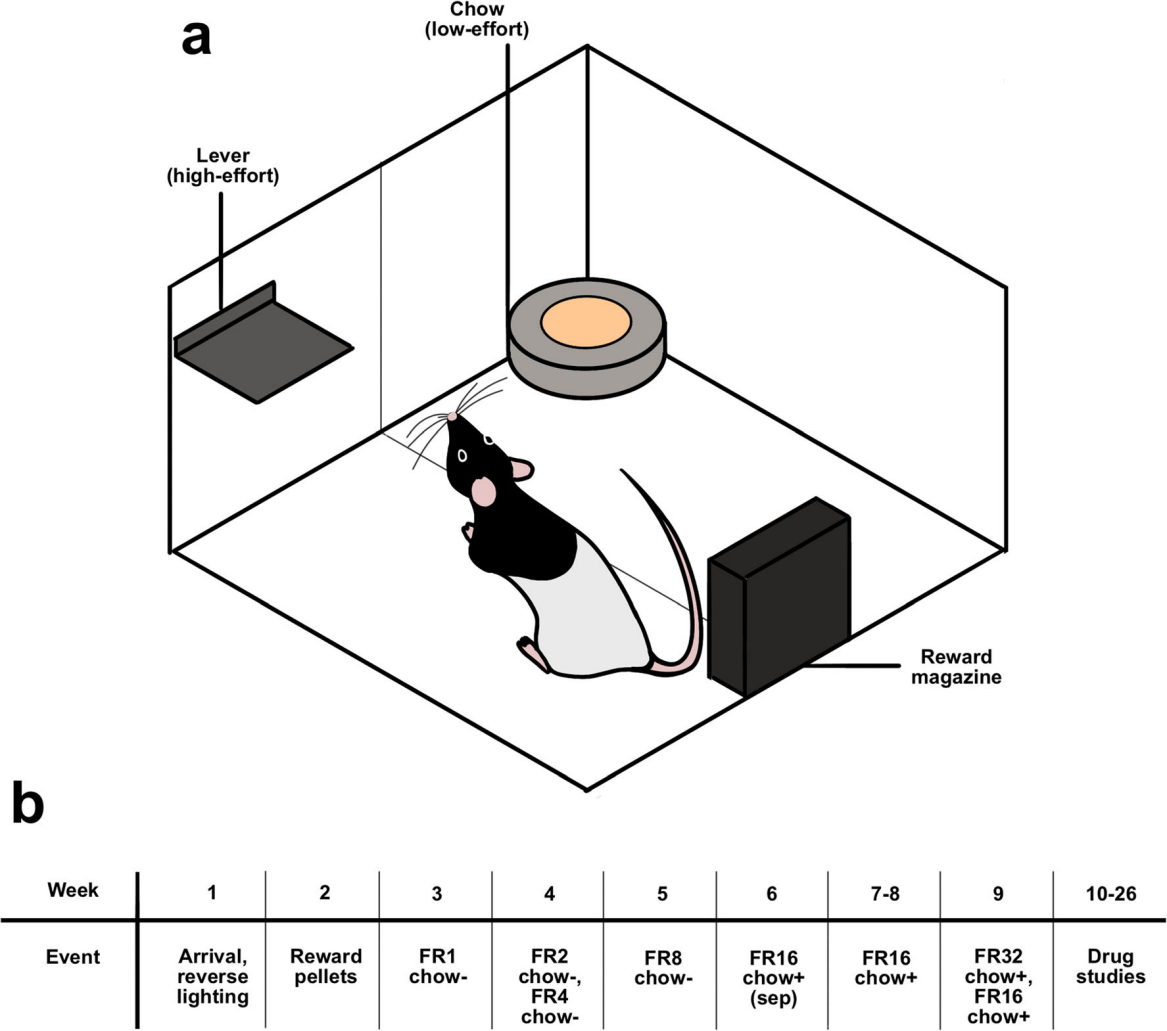
Overview of the EfRT setup and experimental timeline. **a** The EfRT setup inside an operant chamber, with the lever (high effort reward option) and the bowl of chow (low effort reward option) present concomitantly. Reward pellets (high reward) are dispensed from the reward magazine. **b** The experimental timeline, with time in weeks and events that occurred in them. Event refers to either habituation (reverse lighting, reward pellets) or the training procedure (fixed ratio (FR) and chow +/−). On week 6, chow was presented separately

On arrival, rats were habituated to reverse lighting conditions for 1 week followed by 1 week of handling habituation (Fig. 1b). Small amounts of 45 mg sucrose enriched food reward pellets (Test Diet, Sandown Scientific, UK) were introduced to their home cages over several days during the handling habituation week. Behavioural sessions were conducted in standard (length 30 cm, width 25 cm, height 33 cm, grid 3 cm above chamber floor, lever 10 cm above grid) rat operant chambers (Med Associates, Sandown Scientific, UK; KLimbic Software, Conclusive Solutions, UK) during the dark period (approximately 09:00–14:00 h). Lever locations were counter-balanced to either side of the operant chambers. Rats were initially trained to lever press on a continuous reinforcement schedule (30 min sessions, 2 days) to obtain 45 mg sucrose enriched food reward pellets (Test Diet, Sandown Scientific, UK). They were then shifted to the fixed ratio 2 (FR2) schedule (30 min sessions) and were increased gradually until rats reached an FR X schedule. After reaching criteria at FR16, the rats were habituated to bowls of chow (LabDiet, PMI Nutrition International) in turned off operant chambers 1 h after the task, over the course of 3 days. All subsequent sessions had the bowls of chow present during the sessions. The rats were stably performing approximately 1000 lever presses per animal per session over the course of 2 weeks. A test using FR32 resulted in the number of lever presses decreasing, with some animals performing only 200 lever presses and consequently receiving under 10 reward pellets over the course of the session (data not shown). It was therefore determined that FR16 was the optimal difficulty. Following 3 more baseline (nondrug) sessions at FR16, the first drug study commenced. At the end of every session, the rats were immediately removed from the chambers and the behavioural outputs were recorded. Lever presses and chow intake were considered the primary experimental outcomes, with magazine entries, inter-reward intervals, and reward latency considered the secondary outcomes.

#### Experimental procedures

The same group of rats was used for all experiments. All experiments were performed with the experimenter blind to treatment and used a within-subject design, with every rat receiving every drug dose, including the appropriate vehicle (Table 1). The order of doses any given animal received was randomised using a Latin square design. Baseline (nondrug) sessions were conducted on Monday and Thursday. Drug sessions were conducted on Tuesday and Friday. Animals were not run on Wednesday, Saturday, and Sunday. Different drug experiments were performed over the course of 2 weeks, with 1 week of daily baseline sessions (5 days/week) before commencing the next treatment. All drugs were administered by intraperitoneal (IP) injection in a final volume of 1 mL/kg using a modified handling method shown to reduce stress (Stuart and Robinson 2015).

**Table 1:** Experimental treatments used in the EfRT

Experiment sequence	Treatment	Route	Dose (mg/kg)	Vehicle	Administration time before task (min)	*n*
1	Chow pre-feeding	Home cage bowl	-	-	Overnight	16
2	Pellet pre-feeding	Home cage bowl	-	-	120	16
3	Amphetamine	IP	0.1, 0.3, 1.0	Saline 0.9%	15	16
4	Haloperidol	IP	0.01, 0.03, 0.1	DMSO (1%), Cremophor (2%), 0.9% saline (97%)	60	15
5	Venlafaxine	IP	1.0, 3.0, 10.0	Saline 0.9%	30	15
6	Reboxetine	IP	0.1, 0.3, 1.0	Saline 0.9%	30	15
7	Citalopram	IP	1.0, 3.0, 10.0	Saline 0.9%	30	15
8	Ketamine	IP	1.0, 3.0, 10.0	Saline 0.9%	60	15
9	Scopolamine	IP	0.01, 0.03, 0.1	Saline 0.9%	60	15
10	Tetrabenazine	IP	0.1, 0.3, 1.0	DMSO (10%), Cremophor (20%), 0.9% saline (70%)	90	15
11	Amphetamine	IP	0.1, 0.3, 1.0	Saline 0.9%	15	14

*mg/kg*, milligrams per kilogram; *min*, minute; *DMSO*, dimethyl sulfoxide; *IP*, intraperitoneal

#### Experiment 1: effects of different pre-feeding conditions on effort-related choice behaviour in the EfRT

For the pre-feeding studies, ad libitum chow or reward pellets were made available to the rats in their home cages during the chow pre-feeding and the reward pellet pre-feeding experiments, respectively. Chow pre-feeding was conducted overnight prior to the start of the EfRT session. Reward pellet pre-feeding was conducted over 2 h immediately prior to the start of the EfRT session.

#### Experiment 2: pharmacological validation and comparison with previous studies on effort for reward

Doses used were based on previous studies on ABT (Stuart et al. 2017), EfRT (Randall et al. 2012; Yohn et al. 2016a), and other reward processing tasks (Floresco et al. 2008; Bardgett et al. 2009). The psychostimulant, amphetamine, was purchased from Sigma, UK, and dissolved in DMSO (10%), Cremophor (20%), 0.9% saline (70%), which was also used as the vehicle control. Rats received 0–1 mg/kg amphetamine (Floresco et al. 2008; Bardgett et al. 2009) 15 min prior to the start of the session. The D2 antagonist, haloperidol, was purchased from Sigma, UK, and dissolved in DMSO (1%), Cremophor (2%), and 0.9% saline (97%), which was also used as the vehicle control. Rats received 0–0.1 mg/kg haloperidol (Randall et al. 2012) 60 min prior to the start of the session. Tetrabenazine, a vesicular monoamine re-uptake inhibitor, was purchased from Sigma, UK, and dissolved in DMSO (10%), Cremophor (20%), 0.9% saline (70%), which was also used as the vehicle control. Rats received 0–1 mg/kg tetrabenazine (Stuart et al. 2017; Yohn et al. 2016a) 90 min prior to the start of the session. Following the final experiment involving tetrabenazine and a baseline week, a second amphetamine experiment, using the same conditions as the first one had, was carried out to confirm the stability of the EfRT over time.

#### Experiment 3: effects of acute treatment with delayed-onset antidepressants on effort-related choice behaviour in the EfRT

Doses used were based on previous studies in the ABT (Stuart et al. 2013, JBT Hales et al. 2017), and other reward processing tasks (Humpston et al. 2013). Venlafaxine, SNRI, was purchased from Hello Bio, UK, and dissolved in 0.9% saline, which was also used as the vehicle control. Rats received 0–10 mg/kg venlafaxine (Stuart et al. 2013; Humpston et al. 2013) 30 min prior to the session. Reboxetine, NRI, was purchased from Sigma, US, and dissolved in 0.9% saline, which was also used as the vehicle control. Rats received 0–1 mg/kg reboxetine (Stuart et al. 2013; Humpston et al. 2013) 30 min prior to the session. Citalopram, SSRI, was purchased from Hello Bio, UK, and dissolved in 0.9% saline, which was also used as the vehicle control. Rats received 0–10 mg/kg citalopram (Stuart et al. 2013) 30 min prior to the session.

#### Experiment 4: effects of acute treatment with rapid-acting antidepressants on effort-related choice behaviour in the EfRT

Doses used were based on previous studies in the ABT (Stuart et al. 2013, JBT Hales et al. 2017), and other reward processing tasks (Autry et al. 2011, Maeng et al. 2008, Hinchcliffe et al., unpublished; Petryshen et al. 2016). Ketamine, an NMDA receptor antagonist, was purchased from Sigma, US, and dissolved in 0.9% saline, which was also used as the vehicle control. Rats received 0–10 mg/kg ketamine (Stuart et al. 2015; Autry et al. 2011; Maeng et al. 2008) 60 min prior to the session. Scopolamine, a muscarinic antagonist, was purchased from Tocris, UK, and dissolved in 0.9% saline, which was also used as the vehicle control. Rats received 0–0.1 mg/kg scopolamine (Hinchcliffe et al., unpublished; Petryshen et al. 2016) 60 min prior to the experiment.

### Statistical analyses

Lever presses, chow intake, magazine entries, inter-reward intervals, and reward latency were individually analysed using repeated measures ANOVA. Where only two groups were analysed, as in the pre-feeding studies, a paired *t* test was used. When there was a significant ANOVA, sphericity was corrected using the Huynh-Feldt correction and main effects were then further analysed post hoc using pairwise comparisons with Sidak corrections. *α* = 0.05 was used for all statistical tests. Findings were considered trends at *P* < 0.1 and are also discussed. All statistical analyses were performed using SPSS 21.0.0.0 for Windows. GraphPad Prism 7.04 for Windows and Procreate 4.3.9 for iOS were used to draw figures.

## Results

### Experiment 1: effects of different pre-feeding conditions on effort-related choice behaviour in the EfRT

**Fig. 2:**
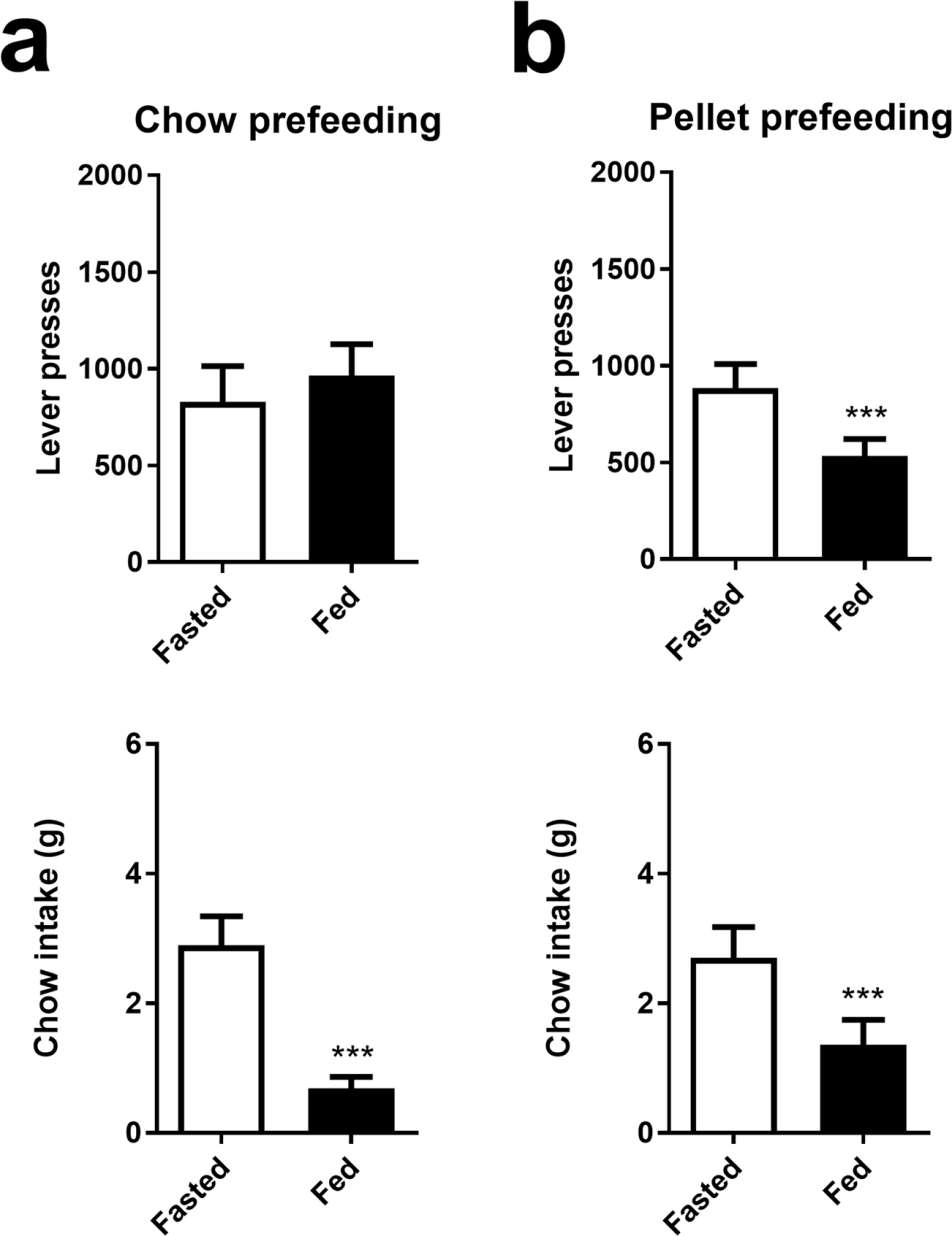
Effects of different pre-feeding conditions on effort-related choice behaviour. **a** Pre-feeding with chow reduced chow intake (bottom) and had no effect on lever presses (top). **b** Pre-feeding with reward pellets reduced both lever presses (top) and chow intake (bottom). Vertical bars indicate the SEM. ***P < 0.001, paired *t* test

Animals exhibited different behavioural patterns depending on the pre-feeding method used. Pre-feeding with chow resulted in reduced chow intake (paired *t* test *P* < 0.01; Fig. 2a) but no effect on lever presses (paired *t* test *P* = 0.104; Fig. 2a). There was an increase in magazine entries (paired *t* test *P* < 0.01; Table 2) and a trend toward reduced inter-reward intervals (paired *t* test *P* = 0.072; Table 2). There was no effect on reward latency (paired *t* test *P* = 0.322; Table 2). In contrast, pre-feeding with reward pellets reduced both chow intake (paired *t* test *P* < 0.001; Fig. 2b) and lever presses (paired *t* test *P* < 0.001; Fig. 2b). There was also a reduction in magazine entries (paired *t* test *P* < 0.05; Table 2) and an increase in inter-reward intervals (paired *t* test *P* < 0.01; Table 2) and reward latency (paired *t* test *P* < 0.05; Table 2).

**Table 2:**
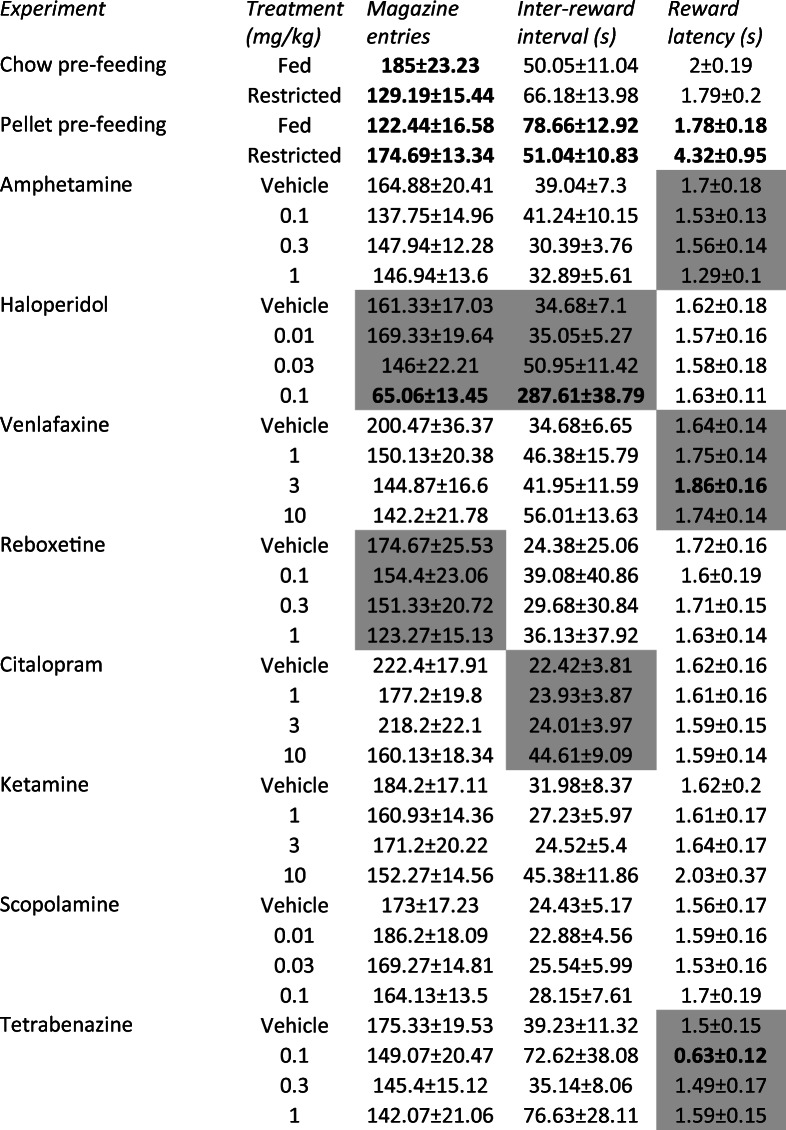
The effects of pre-feeding, acute delayed-onset antidepressants, and acute rapid-acting antidepressants on magazine entries, inter-reward interval, and reward latency in the EfRT

Output values are presented as mean ± SEM. Grey boxes indicate a main effect of dose, repeated measures ANOVA, *P* < 0.05. Bold values indicate Sidak-corrected pairwise comparison effects with vehicle control following main effects of dose, *P* < 0.05. Where only two groups were analysed, bold values indicate *P* < 0.05, paired *t* test

*mg/kg*, milligrams/kilogram; *s*, second

### Experiment 2: pharmacological validation and comparison with previous studies on effort for reward

**Fig. 3:**
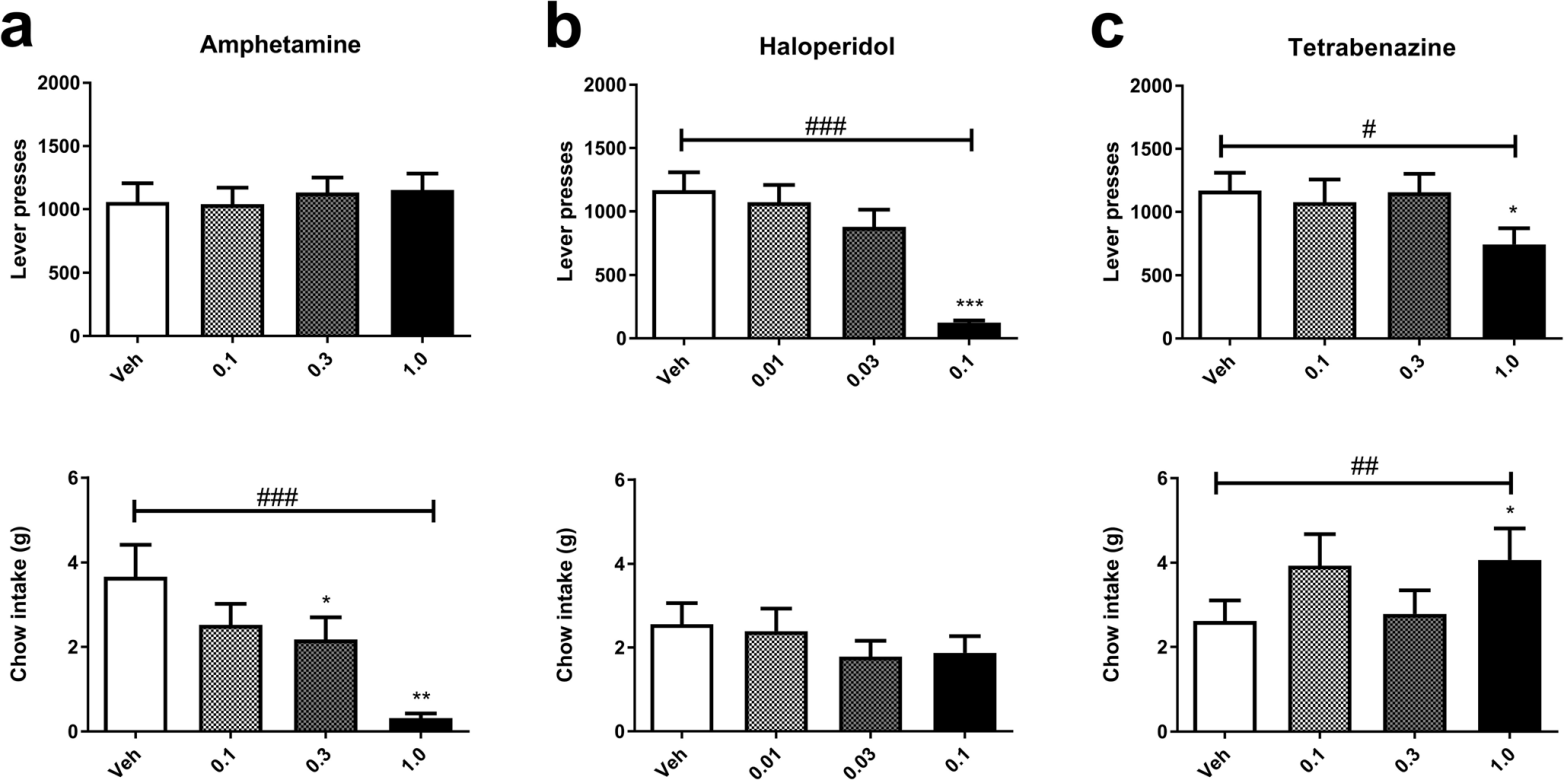
Pharmacological validation and comparison with previous studies on effort for reward. **a** Amphetamine (0.1–1.0 mg/kg) reduced chow intake (bottom) and did not affect lever presses (top). **b** Haloperidol (0.01–0.1 mg/kg) reduced lever presses (top) without affecting chow intake (bottom). **c** Tetrabenazine (0.1–1.0 mg/kg) reduced lever presses (top) and increased chow intake (bottom). Vertical bars indicate the SEM. ^#^*P* < 0.05, ^##^*P* < 0.01, ^###^*P* < 0.001, repeated measures ANOVA. Asterisks immediately above treatment columns represent Sidak-corrected pairwise comparisons with vehicle control following main effects of dose, *P < 0.05, **P < 0.01, ***P < 0.001

There were dissociable effects on high versus low effort choice behaviour dependent on whether dopamine effects were enhanced or inhibited. Amphetamine (0.1–1.0 mg/kg) reduced chow intake (*F*_3, 45_ = 12.69, *P* < 0.001; Fig. 3a) but did not affect lever presses (*F*_3, 45_ = 0.26, *P* = 0.85; Fig. 3a). Post hoc analyses revealed that 0.3 and 1.0 mg/kg reduced chow intake (pairwise comparison *P* < 0.05, *P* < 0.01, respectively; Fig. 3a). There was also a general reduction effect on reward latency (*F*_3, 45_ = 2.987, *P* < 0.05; Table 2), but post hoc tests did not reveal a significant effect. Magazine entries (*F*_3, 45_ = 1.04, *P* = 0.38; Table 2) and inter-reward intervals (*F*_2.24, 33.55_ = 0.592, *P* = 0.577; Table 2) were not affected.

In contrast, administration of haloperidol (0.01–0.1 mg/kg) reduced lever presses (*F*_3, 42_ = 24.188, *P* < 0.001; Fig. 3b) without affecting chow intake (*F*_1.75, 24.5_ = 1.58, *P* = 0.226; Fig. 3b). Post hoc analyses revealed that 0.03 mg/kg reduced lever presses (pairwise comparison *P* < 0.001; Fig. 3b). Haloperidol reduced magazine entries (*F*_3, 42_ = 18.39, *P* < 0.001; Table 2), with a post hoc effect seen for the 0.1 mg/kg dose (pairwise comparison *P* < 0.001; Table 2). Haloperidol also increased inter-reward intervals (*F*_1.13, 15.76_ = 32.51, *P* < 0.001; Table 2), with a post hoc effect seen for the 0.1 mg/kg dose (pairwise comparison *P* < 0.001; Table 2). There was no general effect on reward latency (*F*_2.24, 31.37_ = 0.72, *P* = 0.51; Table 2).

Tetrabenazine administration (0.01–0.1 mg/kg) reduced lever presses (*F*_3, 42_ = 2.891, *P* < 0.05; Fig. 3c) whilst increasing chow intake (*F*_2.03, 28.43_ = 2.891, *P* < 0.005; Fig. 3c). Post hoc analyses revealed that 1 mg/kg reduced lever presses (pairwise comparison *P* < 0.05; Fig. 3c) and increased chow intake (pairwise comparison *P* < 0.05; Fig. 3c), with 0.1 mg/kg tending to increase chow intake (pairwise comparison *P* < 0.1; Fig. 3c). There was also an increase in reward latency (*F*_2.28, 31.90_ = 26.427, *P* < 0.001; Table 2), with the lowest dose of 0.1 mg/kg resulting in a reduction (pairwise comparison *P* < 0.001; Table 2). Tetrabenazine did not affect magazine entries (*F*_3, 42_ = 2.044, *P* = 0.122; Table 2) or inter-reward intervals (*F*_1.19, 16.7_ = 1.086, *P* = 0.326; Table 2).

In order to check whether the sensitivity of the animals had changed over the course of the study, initial amphetamine treatment (0.1–1.0 mg/kg) was repeated at the end of the experimental sequence. The results were similar to those from the first, with a reduction in chow intake (*F*_1.99, 25.92_ = 9.808, *P* < 0.001; Online Resource 1) and no effect on lever presses (*F*_3, 39_ = 1.111, *P* = 0.356; Online Resource 1). Post hoc analysis revealed that the 1.0 mg/kg dose produced a reduction in chow intake (pairwise comparison *P* < 0.05; Online Resource 1) and the 0.3 dose tended to reduce chow intake (pairwise comparison *P* < 0.1; Online Resource 1). Amphetamine induced a reduction in reward latency (*F*_1.24, 16.08_ = 5.752, *P* < 0.05; Online Resource 2), but post hoc tests did not reveal any dose-specific effects. There was no effect on magazine entries (*F*_1.51, 19.59_ = 0.418, *P* = 0.608; Online Resource 2) or inter-reward intervals (*F*_1.33, 17.35_ = 0.949, *P* = 0.371; Online Resource 2). Baseline levels of chow intake numerically increased but this was not significant when the two studies were compared directly (paired *t* test *P* = 0.069; Online Resource 1). There was a significant increase in baseline lever presses when the two studies were directly compared (paired *t* test *P* < 0.01; Online Resource 1), but this did not influence the main effects of amphetamine, which were similar for both studies.

### Experiment 3: effects of acute treatment with delayed-onset antidepressants on effort-related choice behaviour in the EfRT

**Fig. 4:**
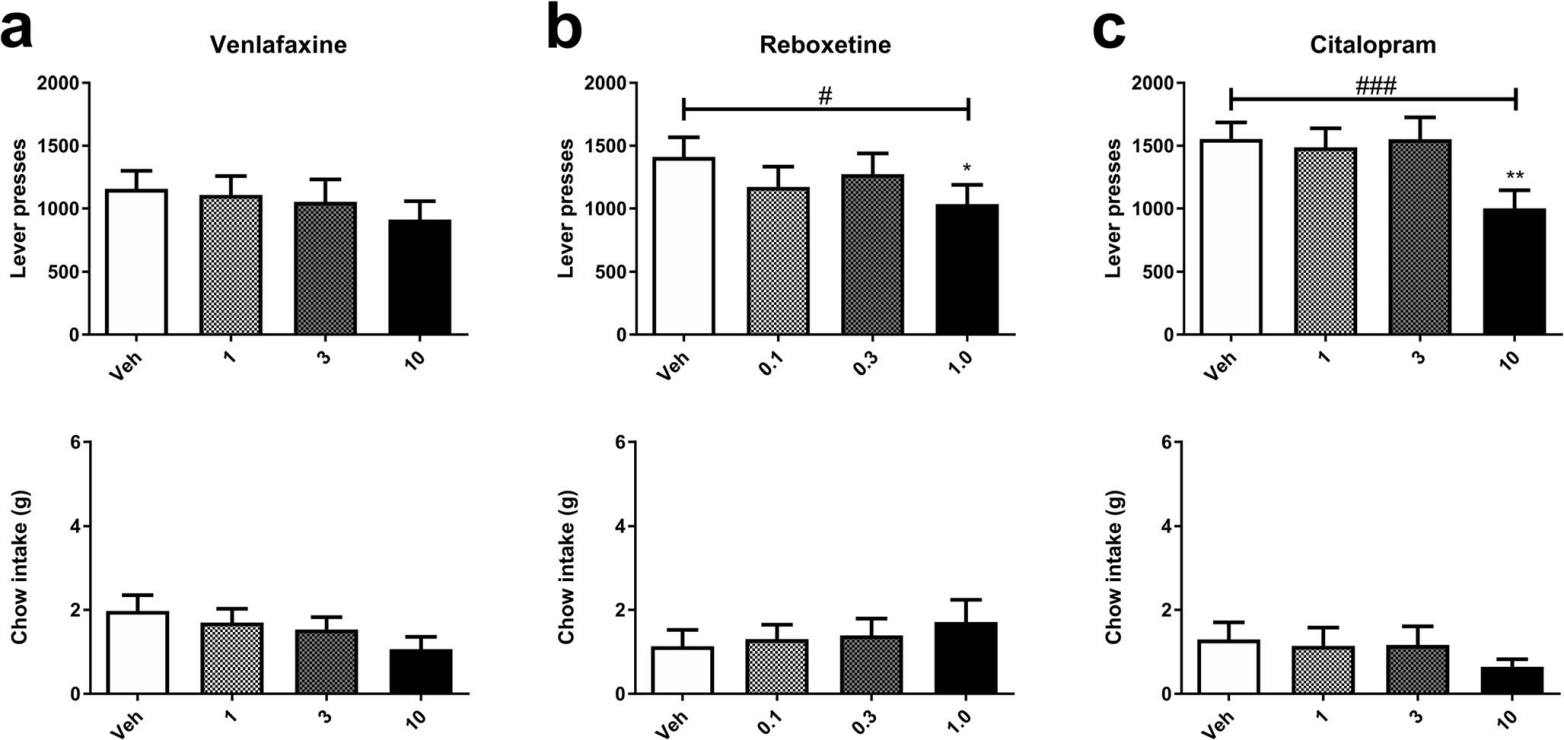
Effects of acute treatment with delayed-onset antidepressants on effort-related choice behaviour in the EfRT. **a** Venlafaxine (1.0–10.0 mg/kg) had no effect on chow intake (bottom) or lever presses (top). **b** Reboxetine (0.1–1.0 mg/kg) reduced lever presses (top) without affecting chow intake (bottom). **c** Citalopram (1.0–10.0 mg/kg) reduced lever presses (top) and did not affect chow intake (bottom). Vertical bars indicate the SEM. ^#^*P* < 0.05, ^##^*P* < 0.01, ^###^*P* < 0.001, repeated measures ANOVA. Asterisks immediately above treatment columns represent Sidak-corrected pairwise comparisons with vehicle control following main effects of dose, *P < 0.05, **P < 0.01, ***P < 0.001

Acute treatment with delayed-onset ADs did not increase high effort choice behaviour but did result in reductions in one or both reward choices at higher doses suggesting effects on measures of motivation. Venlafaxine treatment (1–10 mg/kg) increased reward latency (*F*_3, 42_ = 4.752, *P* < 0.01; Table 2), with post hoc tests revealing an effect at 3 mg/kg (pairwise comparison *P* < 0.05; Table 2). Venlafaxine tended to reduce chow intake (*F*_2.29, 32.03_ = 3.123, *P* = 0.051; Fig. 4a) and magazine entries (*F*_1.94, 27.13_ = 3.318, *P* = 0.053; Table 2). There was no effect on lever presses (*F*_1.98, 27.78_ = 1.39, *P* = 0.267; Fig. 4a) or on inter-reward intervals (*F*_2.064, 28.90_ = 1.174, *P* = 0.325; Table 2).

Administration of reboxetine (0.1–1.0 mg/kg) reduced lever presses (*F*_3, 39_ = 3.787, *P* < 0.05; Fig. 4b), with post hoc analysis indicating that 1.0 mg/kg produced a reduction in lever presses (pairwise comparison *P* < 0.05; Fig. 4b). Although there was no general effect on chow intake (*F*_1.85, 24.03_ = 0.902, *P* = 0.412; Fig. 4b), inter-reward intervals (*F*_1.60, 20.82_ = 1.338, *P* = 0.279; Table 2), or on reward latency (*F*_3, 42_ = 0.659, *P* = 0.582; Table 2), reboxetine reduced magazine entries (*F*_3, 36_ = 2.979, *P* < 0.05; Table 2), but post hoc analysis did not reveal any specific dose effects.

Citalopram treatment (1–10 mg/kg) also reduced lever presses (*F*_3, 42_ = 8.261, *P* < 0.001; Fig. 4c), with post hoc analysis revealing a reduction in lever presses at 10 mg/kg (pairwise comparison *P* < 0.001; Fig. 4c). There was no effect on chow intake (*F*_3, 42_ = 1.941, *P* = 0.138; Fig. 4c), magazine entries (*F*_3, 42_ = 2.208, *P* = 0.103; Table 2), or reward latency (*F*_3, 42_ = 0.104, *P* = 0.957; Table 2). There also was a general effect on inter-reward intervals (*F*_1.293, 18.10_ = 5.509, *P* < 0.05; Table 2), with no specific effects revealed by post hoc test.

### Experiment 4: effects of acute treatment with rapid-acting antidepressants on effort-related choice behaviour in the EfRT

**Fig. 5:**
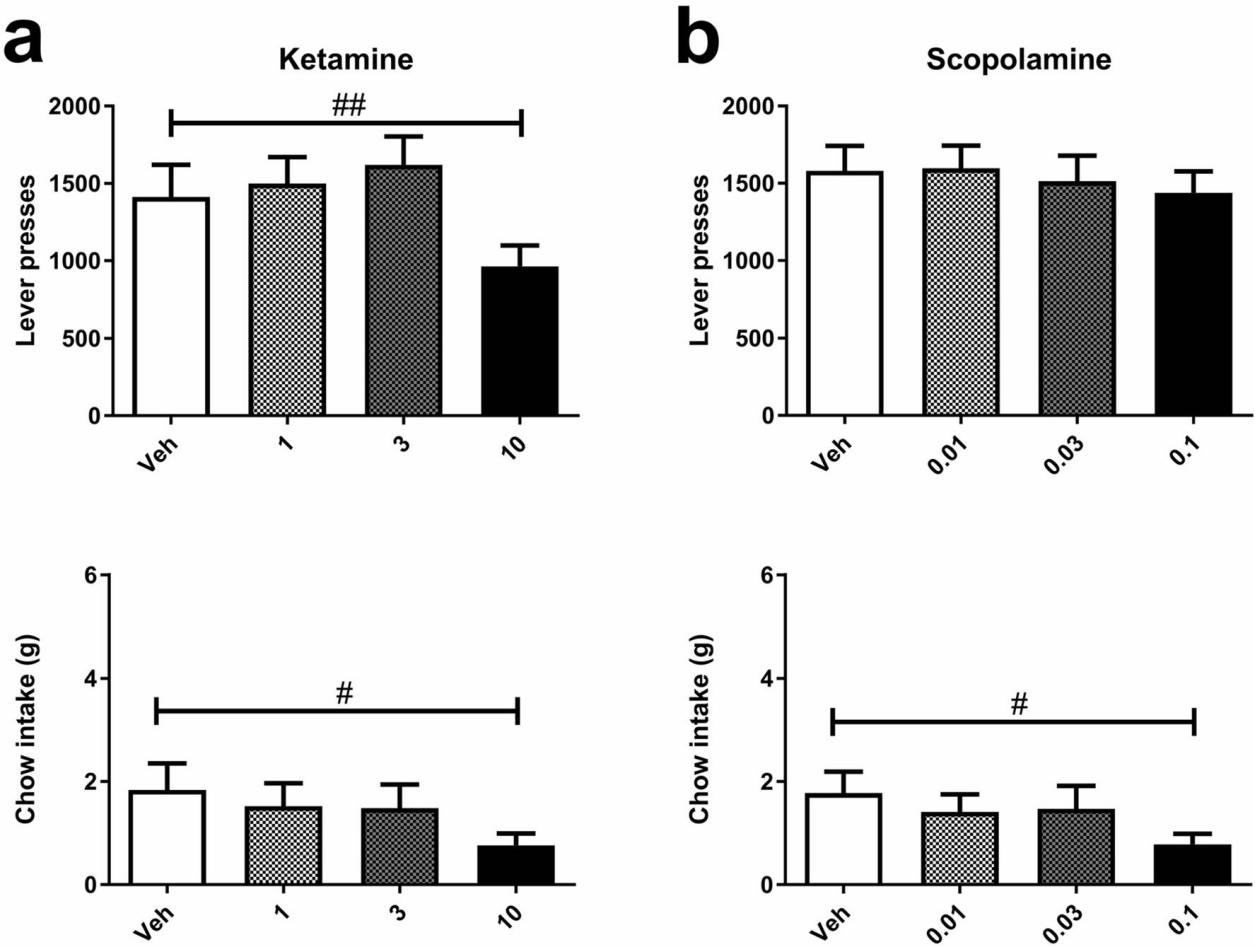
Effects of acute treatment with rapid-acting antidepressants on effort-related choice behaviour in the EfR task. **a** Ketamine (1.0–10.0 mg/kg) reduced both lever presses (top) and chow intake (bottom). **b** Scopolamine (0.01–0.1 mg/kg) reduced chow intake (bottom) without affecting lever presses (top). Vertical bars indicate the SEM. ^#^*P* < 0.05, ^##^*P* < 0.01, ^###^*P* < 0.001, repeated measures ANOVA. Asterisks immediately above treatment columns represent Sidak-corrected pairwise comparisons with vehicle control following main effects of dose, *P < 0.05, **P < 0.01, ***P < 0.001

Neither ketamine nor scopolamine exhibited any specific effects on effort-related choice behaviour. Both drugs changed variables linked to motivation but there were differences in exact effects between drug classes. Ketamine (1–10 mg/kg) caused a general impairment and reduced both lever presses (*F*_3, 42_ = 6.529, *P* < 0.001; Fig. 5a) and chow intake (*F*_1.93, 27.02_ = 4.694, *P* < 0.05; Fig. 5a). Post hoc analysis did not find any dose-specific differences but there was a tendency to reduce chow intake at 10 mg/kg (pairwise comparison *P* < 0.1; Fig. 5a). There was also a tendency for ketamine to increase inter-reward intervals (*F*_3, 42_ = 2.638, *P* < 0.1; Table 2), but with no effect on magazine entries (*F*_3, 42_ = 0.1313, *P* = 2.83; Table 2) or reward latency (*F*_1.286, 18.0_ = 1.056, *P* = 0.338; Table 2).

Scopolamine administration (0.01–0.1 mg/kg) resulted in a reduction in chow intake (*F*_3, 42_ = 3.375, *P* < 0.05; Fig. 5b), but post hoc tests did not reveal any individual dose effects. There was a general trend for scopolamine to increase reward latency (*F*_3, 42_ = 2.636, *P* = 0.062; Table 2); however, scopolamine did not affect lever presses (*F*_3, 42_ = 1.32, *P* = 0.283; Fig. 5b), magazine entries (*F*_2.05, 28.75_ = 1.08, *P* = 0.354; Table 2), or inter-reward intervals (*F*_2.01, 28.17_ = 1.363, *P* = 0.272; Table 2).

## Discussion

In these studies, we observe dissociable effects with dopaminergic drug treatments and the vesicular monoamine transport inhibitor consistent with previous studies. However, we found no evidence that acute treatments with conventional delayed-onset or rapid-acting ADs produce a behavioural shift toward the high value-high effort option in our EfRT. In fact, in most cases, the treatments induced a reduced willingness to engage in the high effort choice behaviour when doses of the drugs tested were increased. This suggests that acute modulation of effort-related choice behaviour in this task is not associated with the antidepressant and anti-anhedonic effects of these treatments. Interestingly, these data contrast with our previous findings (Stuart et al. 2015; Hales et al. 2017) where these same treatments induced positive affective biases in measures of reward learning and memory or decision-making. Despite their clear temporal differences in efficacy in patients, there was no difference between delayed-onset ADs and rapid-acting ADs in this EfRT with both classes of AD reducing high effort-high reward choices at higher doses. The effects we observed are similar to those in other reports with delayed-onset ADs in normal animals and add to the literature for this task with new data for rapid-acting ADs. These studies used comparable doses to those used in our affective bias tasks but doses which we have previously found to affect reward-related affective biases did not have any effects in this task suggesting different neuropsychological mechanisms are involved. The following discussion considers these data in the context of previous studies into effort-related choice behaviour and the wider discussion relating to reward deficits in MDD.

Consistent with previous studies (Salamone et al. 1991; Randall et al. 2012; Yohn et al. 2016a), the D2 antagonist, haloperidol, decreased lever presses and magazine entries whilst increasing inter-reward intervals but with no effect on chow intake. This suggests that haloperidol reduced willingness to exert effort whilst not affecting appetite. In the present study, the psychostimulant, amphetamine, reduced chow intake and decreased reward latency. These effects are consistent with the anorectic and locomotive properties of the drug (Bardgett 1999), although the effect on reward latency could be due to increased reward seeking. Despite amphetamine resulting in an increase in the high effort choice in a barrier climbing task (Bardgett et al. 2009) and in the human EEfRT (Wardle et al. 2011), in our EfRT, amphetamine did not produce an increase in lever presses. In an effort-discounting task, Floresco et al. (2008) reported a small increase in effortful behaviour at low doses of amphetamine, but a dramatic decrease at 0.5 mg/kg. These discrepancies may reflect differences among the tasks, but it is unlikely to be due to a ceiling effect in our task as many animals performed well in excess of 1000 lever presses during baseline sessions (data not shown). The human EEfRT has both an effort and a cognitive element in that participants are required to integrate reward probability with effort, and therefore, the human EEfRT is somewhat different to the rat EfRT used here. A progressive ratio version of the EfRT, due to the higher ratio trials also requiring more time, may produce results more similar to the ones shown by Floresco et al. (2008) but this was not tested here. When the reward schedule is not fixed but instead a progressive ratio of the task is used, dopaminergic drugs such as lisdexamfetamine (Yohn et al. 2016c), PRX-14040 (Yohn et al. 2016b), and CE-123 (Rotolo et al. 2019) appear to potentiate high effort responding. It is possible the progressive ratio schedule results in reduced levels of effortful behaviour when chow is also present, as indicated by the relatively low numbers of lever presses, thus providing a better baseline for the assessment of whether drugs increase motivation. Depletion of monoamine transmitters using the vesicular monoamine re-uptake inhibitor, tetrabenazine, produced a shift from willingness to exert effort for reward to enhanced chow intake. These findings are similar to those reported by Yohn et al. (2016a) and Nunes et al. (2013) in the FR5/chow feeding task and in the T-maze barrier choice task (Yohn et al. 2015). The parallels between the amphetamine-driven reduction in chow intake and the tetrabenazine-driven increase in chow intake as well as a decrease in lever presses point to a monoamine-dependent effect on motivation that can be shifted pharmacologically up or down. In order to check that animal’s performance in the task remained consistent throughout the course of the study, the final experiment re-examined the effects of amphetamine. Consistent with the first amphetamine study, a reduction in chow intake but not lever press responses was observed, suggesting animals remained sensitive at least to the dopaminergic manipulation despite repeated testing. These pharmacological studies suggest that EfRT is sensitive to changes in dopaminergic transmission and that effects on choice behaviour can be dissociated from changes in appetite and general changes in motivation for food.

In order to explore effects of appetite further, we tested both chow and pellet pre-feeding. Chow pre-feeding produced a marked reduction in chow intake and an increase in magazine entries but without affecting lever presses. Pellet pre-feeding reduced most measures, including lever presses, chow consumption, magazine entries, and reward latency, with the inter-reward interval being increased. These findings expand upon those of Salamone et al. (1991) and Randall et al. (2012), where the authors reported a decrease in both chow intake and lever presses in the FR5/chow feeding task and the PROG/chow feeding task following pre-feeding with chow and reward pellets together. In the study by Thompson et al. (2017), pre-feeding with reward pellets in the PROG/chow task reduced both appetite and motivation, indicating the devaluation of the reward and consistent with our EfRT results.

Although studies in the ABT have previously found that delayed-onset ADs increase choices for reward-paired cues learnt following acute treatment, the same doses and treatment protocol used in the EfRT did not enhance high effort choices and had no effect on chow intake. In fact, both reboxetine and citalopram reduced lever responses at the highest doses. Reboxetine and venlafaxine also reduced magazine entries whilst citalopram increased the inter-trial interval, and venlafaxine increased reward latency. Overall, this profile of effects suggests acutely administered delayed-onset ADs made the animals less interested in the high effort-high reward option. Similarly, desipramine, fluoxetine, and bupropion, also delayed-onset ADs, when given acutely and alone, failed to increase high effort choice in the FR5/chow feeding task (Yohn et al. 2016a). Bupropion did attenuate the tetrabenazine-induced reduction in high effort choices, unlike any other typical delayed-onset AD tested (Nunes et al. 2013; Yohn et al. 2016a). As mentioned earlier, fluoxetine infusions into the lateral habenula failed to inhibit synchronous burst firing that was found to drive the depressed phenotype seen in the SPT, forced swim test, and the real-time place aversion task (Yang et al. 2018). Additionally, Hosking et al. (2015) showed that the administration of atomoxetine, another delayed-onset AD, failed to increase high effort choice behaviour in the rat cognitive effort and the effort-discounting tasks. More generally, previous operant tasks investigating delayed-onset ADs have also observed reduced motivation to respond for reward at high doses (Anderson et al. 2013; Humpston et al. 2013; Rygula et al. 2014). For example, Humpston et al. (2013) observed reduced premature responding and increased omissions with acute AD treatment in the 5-choice serial reaction time task, which may indicate reduced motivation, although sedative drugs may also have similar effects through non-specific mechanisms rather than a specific effect on motivation. These reports of either no delayed-onset AD effects on motivation or indeed of motivation impairment in different rodent tasks, as well as our results here, are consistent with the human findings that delayed-onset ADs either lack efficacy in terms of their effects on motivational impairments or worsen the said impairments (Stenman and Lilja 2013; Fava et al. 2014; Rothschild et al. 2014)

In this study, rapid-acting ADs also failed to increase lever presses, but only ketamine, and not scopolamine, reduced lever presses at the highest dose. Seeing as both rapid-acting ADs reduced chow intake at higher doses, whilst ketamine increased the inter-trial interval and scopolamine increased reward latency, it suggests acutely administered rapid-acting ADs, like delayed-onset ADs, decrease willingness to exert effort for higher reward. Together, these findings point to the lack of dissociation between delayed-onset and rapid-acting ADs, at least in terms of their effects on motivation. Furthermore, these findings suggest that, whilst EfRT is sensitive to acute dopaminergic manipulations, it is not sensitive to acute noradrenergic, serotonergic, glutamatergic, and muscarinic manipulations at the low to medium doses which were found to be effective in the affective bias test, as the impairing effects seen in this study only arose at the higher doses. There remains the possibility that these manipulations may rescue an induced phenotype, such as achieved with tetrabenazine-induced deficit (Nunes et al. 2013; Yohn et al. 2016a). The lack of effect of these rapid-acting ADs contrasts with results from affective bias tasks where low doses of ketamine attenuate previously learnt, negative reward-related biases (Stuart et al. 2015) and induce more optimistic reward-related decision-making (Hales et al. 2017). Previous clinical studies have found that ketamine ameliorated anhedonia in patients with treatment-resistant depression and treatment-resistant bipolar depression (Murrough et al. 2013; Lally et al. 2014) suggesting the reward deficits affected in the EfRT differ from those quantified in the affective bias tasks and in patients. Taken together, these findings suggest that delayed-onset ADs do not produce a positive shift in motivation in normal animals, but neither do the rapid-acting ADs, ketamine and scopolamine, with the possibility remaining that they could rescue a depressive phenotype.

In summary, our findings in this EfRT confirm conclusions from previous studies and suggest this task is sensitive to dopaminergic modulation of effort-related choice behaviours and these can be differentiated from general motivation or appetite changes. In these studies, and consistent with previous work with delayed-onset ADs, we did not observe any specific effects on effort-related choice behaviour. We extend this by also testing rapid-acting ADs but found no evidence for any specific effects. These findings add further to the discussion relating to the different aspects of reward-related behaviour and how they may be altered in MDD and respond to AD treatment. More broadly, and in combination with findings from previous studies using delayed-onset and rapid-acting ADs, it is likely that the affective bias phenotype seen in the ABT is driven by changes in reward-related cognition and not by reward sensitivity, motivation, and effort-related choice (Slaney et al. 2018). The current EfRT study focused on acute effects using doses matched to previous ABT studies, and our findings do not preclude the possibility that motivational changes may be observed following chronic administration. Furthermore, this study only looked at effects in female rats. Although previous work in the ABT has shown similar effects with these treatments in males and females, it is not necessarily the case that the findings here would be the same in male subjects. Whilst depression is more prevalent in females (Kuehner 2017), future studies looking at EfRT in male rats or ideally studies directly comparing both sexes would be useful. In order to expand on the current findings even further, future studies on EfRT should also investigate the effects of delayed-onset and rapid-acting ADs following the induction of a depressive phenotype, as well as the effects of chronic AD administration.

## Electronic supplementary material

Online resource 1 (PDF 113 kb)

Online resource 2 (PDF 129 kb)

## Abbreviations

MDD: Major depressive disorder
AD: Antidepressant drugs
ABT: Affective bias test
EfRT: Effort for reward task
JBT: Judgement bias task
DSM5: Diagnostic and Statistical Manual of Mental Disorders 5
SPT: Sucrose preference test
EEfRT: Effort expenditure for rewards task
NMDA: N-Methyl-d-aspartate
