# Supplementary material for: Comparison of acute treatment with delayed-onset versus rapid-acting antidepressants on effort-related choice behaviour
Source: Psychopharmacology (Berl). 2020 May 20;237(8):2381–94. doi: 10.1007/s00213-020-05541-9 (PMC7351874; doi:10.1007/s00213-020-05541-9)
Supplement: Supplementary file 1 — (PDF 113 kb) [file 213_2020_5541_MOESM1_ESM.pdf]

Online resource 1

Comparison of acute treatment with delayed-onset versus rapid-acting antidepressants on effort-related choice behaviour

Psychopharmacology

Simonas Griesius, Jack R Mellor, Emma SJ Robinson

University of Bristol

emma.s.j.robinson@bristol.ac.uk

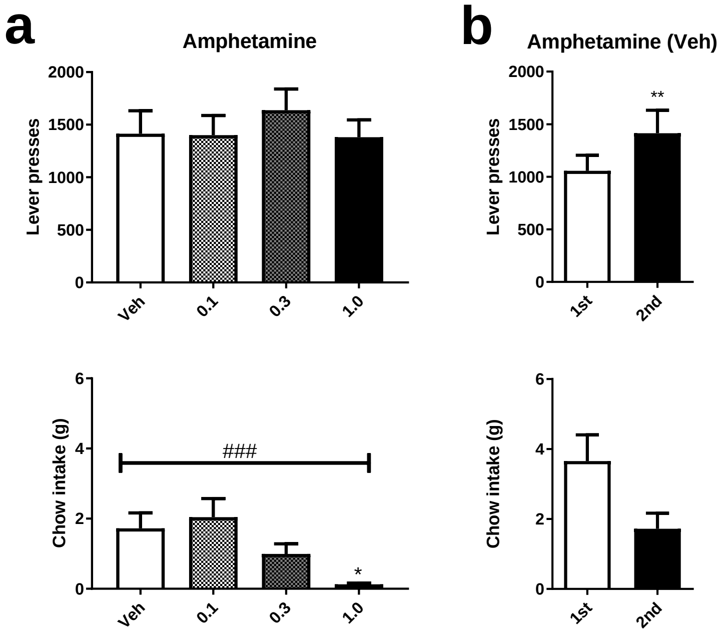

Fig. S1 Pharmacological validation of EfRT stability. a) Amphetamine (0.1 - 1.0 mg/kg) reduced chow intake (bottom) and did not affect lever presses (top). b) Comparison of amphetamine vehicle treatment across the 1st and 2nd amphetamine experiments. Lever presses (top) were increased, whilst chow intake (bottom) tended to decrease in the 2nd experiment. Vertical bars indicate the SEM. # P < 0.05, ## P < 0.01, ### P < 0.001, repeated measures ANOVA. Asterisks immediately above treatment columns represent Sidak-corrected pairwise comparisons with vehicle control following main effects of dose or paired t-tests, as appropriate, * P < 0.05, ** P < 0.01, *** P < 0.001
