# Supplementary material for: Comparison of acute treatment with delayed-onset versus rapid-acting antidepressants on effort-related choice behaviour
Source: Psychopharmacology (Berl). 2020 May 20;237(8):2381–94. doi: 10.1007/s00213-020-05541-9 (PMC7351874; doi:10.1007/s00213-020-05541-9)
Supplement: Supplementary file 2 — (PDF 129 kb) [file 213_2020_5541_MOESM2_ESM.pdf]

Online resource 2

Comparison of acute treatment with delayed-onset versus rapid-acting antidepressants on effort-related choice behaviour

Psychopharmacology

Simonas Griesius, Jack R Mellor, Emma SJ Robinson

University of Bristol

emma.s.j.robinson@bristol.ac.uk

Table S1 The effects of amphetamine (second administration at the end of the experimental sequence) on magazine entries, inter-reward interval, and reward latency in the EfR task

Experiment	*Treatment (mg/kg)*	*Magazine entries*	*Inter-reward interval (s)*	*Reward latency (s)*
Amphetamine	Vehicle	204.21±30.8	39.39±13.61	2.48±0.42
	0.1	203.64±33.01	37.24±14.77	1.46±0.16
	0.3	224.79±38.55	22.73±3.85	1.46±0.19
	1	195.43±22.98	30.03±7.6	1.26±0.12

Output values are presented as mean ± SEM. Grey boxes indicate a main effect of dose, repeated measures ANOVA, $P < 0.05$

mg/kg milligrams/kilogram

s second
